# Enteropathogenic *Escherichia coli* regulates host-cell mitochondrial morphology

**DOI:** 10.1080/19490976.2022.2143224

**Published:** 2022-12-08

**Authors:** Jennifer Lising Roxas, Shylaja Ramamurthy, Katie Cocchi, Ilga Rutins, Anusha Harishankar, Al Agellon, John Scott Wilbur, Gresa Sylejmani, Gayatri Vedantam, V.K. Viswanathan

**Affiliations:** a School of Animal and Comparative Biomedical Sciences, University of Arizona, Tucson, AZ, USA; b Department of Immunobiology, University of Arizona, Tucson, AZ, USA; c BIO5 Institute for Collaborative Research, University of Arizona, Tucson, AZ, USA; d Research Service, Southern Arizona VA Healthcare System, Tucson, AZ, USA

**Keywords:** Enteropathogenic *Escherichia coli*, EPEC, EspZ, EspH, FIS1, mitophagy, mitochondrial fission, mitochondrial fusion

## Abstract

The diarrheagenic pathogen enteropathogenic *Escherichia coli* is responsible for significant childhood mortality and morbidity. EPEC and related attaching-and-effacing (A/E) pathogens use a type III secretion system to hierarchically deliver effector proteins into host cells and manipulate epithelial structure and function. Subversion of host mitochondrial biology is a key aspect of A/E pathogen virulence strategy, but the mechanisms remain poorly defined. We demonstrate that the early-secreted effector EspZ and the late-secreted effector EspH have contrasting effects on host mitochondrial structure and function. EspZ interacts with FIS1, a protein that induces mitochondrial fragmentation and mitophagy. Infection of epithelial cells with either wildtype EPEC or an isogenic *espZ* deletion mutant (Δ*espZ*) robustly upregulated FIS1 abundance, but a marked increase in mitochondrial fragmentation and mitophagy was seen only in Δ*espZ-*infected cells. FIS1-depleted cells were protected against Δ*espZ-*induced fission, and EspZ-expressing transfected epithelial cells were protected against pharmacologically induced mitochondrial fission and membrane potential disruption. Thus, EspZ interacts with FIS1 and blocks mitochondrial fragmentation and mitophagy. In contrast to WT EPEC, Δ*espH-*infected epithelial cells had minimal FIS1 upregulation and exhibited hyperfused mitochondria. Consistent with the contrasting impacts on organelle shape, mitochondrial membrane potential was preserved in Δ*espH-*infected cells, but profoundly disrupted in Δ*espZ-*infected cells. Collectively, our studies reveal hitherto unappreciated roles for two essential EPEC virulence factors in the temporal and dynamic regulation of host mitochondrial biology.

## Plain Language Summary

Bacterial pathogens strategically manipulate host cell structures and functions during the process of colonization and expansion, and this eventually contributes to disease symptoms. The diarrhea-causing pathogen enteropathogenic *Escherichia coli* (EPEC) secretes proteins into host cells to alter their behavior. Two secreted proteins, EspZ and EspH, were previously shown to be essential for causing disease in animal models. In this study, we demonstrate that interplay between EspZ/EspH and host factors modulates the structure and function of host cell mitochondria. Among their various roles, mitochondria generate energy, produce important biomolecules, and protect cells from damage. EPEC infection of epithelial cells results in increased abundance of a key mitochondrial outer-membrane protein, FIS1. FIS1 plays a housekeeping role by breaking down unhealthy mitochondria and targeting them for elimination from cells. In the early stages of infection, EspZ interacts with FIS1 and blocks its action, thereby protecting the host mitochondrial network and consequently, enhancing host cell viability. Our studies are consistent with a model wherein EspZ-dependent preservation of mitochondrial integrity early in infection allows for bacterial colonization. Later in infection, however, EspH-dependent increase in FIS1 results in significant mitochondrial fragmentation and host cell death; this likely facilitates pathogen dispersal. Taken together, EspZ and EspH dynamically impact host biology, and consequently, infection outcomes. Overall, an appreciation of the mechanisms by which EspZ and EspH manipulate host cells could eventually lead to host-directed interventions for EPEC diarrhea, which is currently not vaccine-preventable.

## Introduction

Enteropathogenic *Escherichia coli* (EPEC) is a leading cause of juvenile diarrheal disease and mortality. EPEC belongs to a family of organisms, known as attaching and effacing (A/E) pathogens that intimately attach to intestinal epithelial cells and efface brush-border microvilli.^1–3^ A/E pathogens use a type III secretion system (T3SS) to inject specific ‘effector’ proteins into host cells in a hierarchical manner.^3^ Together, the T3SS and effector proteins subvert epithelial cell structure and function, and contribute to virulence.^4–10^ The mitochondria of intestinal epithelial cells are key targets of A/E pathogen virulence factors.^11^

Mitochondrial shape, function and positioning are dynamically regulated in eukaryotic cells.^12^ The processes of fusion, fission and mitophagy regulate mitochondrial shape in response to cellular cues, including stress. Fission facilitates organelle distribution during mitosis. It also serves to demarcate and dispose of damaged mitochondria via mitophagy wherein defective organelles are enclosed in a double membrane, and eliminated via a lysosome-dependent pathway.^13^ On the other hand, mitochondrial fusion alleviates cellular stress by combining the contents of partially damaged organelles.^13^ Overall, impaired fusion promotes mitochondrial fragmentation (which is correlated with lower respiratory activity), while inhibition of fission leads to fusion of adjacent mitochondria resulting in fused networks that have increased numbers of cristae, and that are optimal for ATP synthesis.^14^

EPEC and related pathogens manipulate host mitochondrial fusion and fission, but the underlying mechanisms remain undefined. Jejunal and ileal biopsies from EPEC-infected children revealed enlarged and disorganized mitochondria.^15^ Colonocytes of mice infected with the murine A/E pathogen *Citrobacter rodentium* had swollen mitochondria with a disrupted matrix and dense inclusion bodies, and displayed perinuclear clustering of the mitochondrial network.^16^ These changes were attributed to the mitochondria-targeted effector protein Map since isogenic *map* deletion strains (Δ*map*) were impaired for inducing such alterations in the mouse colon. EPEC infection of HeLa cells resulted in Map-dependent mitochondrial swelling and dysfunction,^17^ and expression of EPEC Map in *Saccharomyces cerevisiae* caused mitochondrial fragmentation.^18^ In a recent study, Map was shown to trigger DRP1-dependent mitochondrial fission in bovine mammary epithelial cells. Consistent with the structural changes noted above, Map disrupted mitochondrial membrane potential (ΔΨm), perturbed cell respiratory functions, and caused host cell apoptosis.^16,18,19^

It is clear, however, that there is further complexity to A/E pathogen-induced mitochondrial alterations, potentially involving contributions from other effector molecules.^11^ The secreted protein EspF, like Map, localizes to the mitochondria, disrupts ΔΨm, and initiates intrinsic apoptosis,^20,21^ although it does not overtly alter organelle morphology.^18^ Another effector protein, EspH, inhibits Rho GTPases, disrupts the cytoskeleton and cytokeratin networks, and contributes to host cell death.^22–25^ While it is well recognized that the cytoskeleton plays important roles in regulating mitochondrial morphology and function,^26^ it is not known if EspH has any impact on the host mitochondrial network.

Interestingly, EPEC infection of TC-7 cells, which are sub-clones of Caco-2 intestinal epithelial-origin cells, caused the formation of fused, toroidal mitochondria;^27^ similar morphology was also observed in neuronal cells overexpressing the fusion protein MFN2 (which promotes fusion), or the dominant negative form of DRP1 (a protein required for mitochondrial fission).^28^ This suggests that EPEC effector proteins could be targeting both mitochondrial fission and fusion processes in host epithelial cells.

We previously showed that EspZ, a 98–100 amino acid protein that localizes to host cell mitochondria, curtailed intrinsic apoptosis induced by the broad-spectrum protein kinase inhibitor staurosporine.^29^ Since staurosporine triggers mitochondrial fragmentation, we hypothesized that EspZ inhibits mitochondrial fission and/or promotes mitochondrial fusion. In this work, we identified the mitochondrial fission protein FIS1 as an EspZ interactor, showed that increased FIS1 abundance was primarily driven by the effector EspH, and defined the impacts of EspZ and EspH on host cell mitochondrial morphology. Our studies highlight novel roles for the EPEC effector EspZ and EspH in dynamically regulating mitochondrial structure and function.

## Results

### EspZ interacts with FIS1 and localizes to the mitochondrial membrane

**Figure 1.**
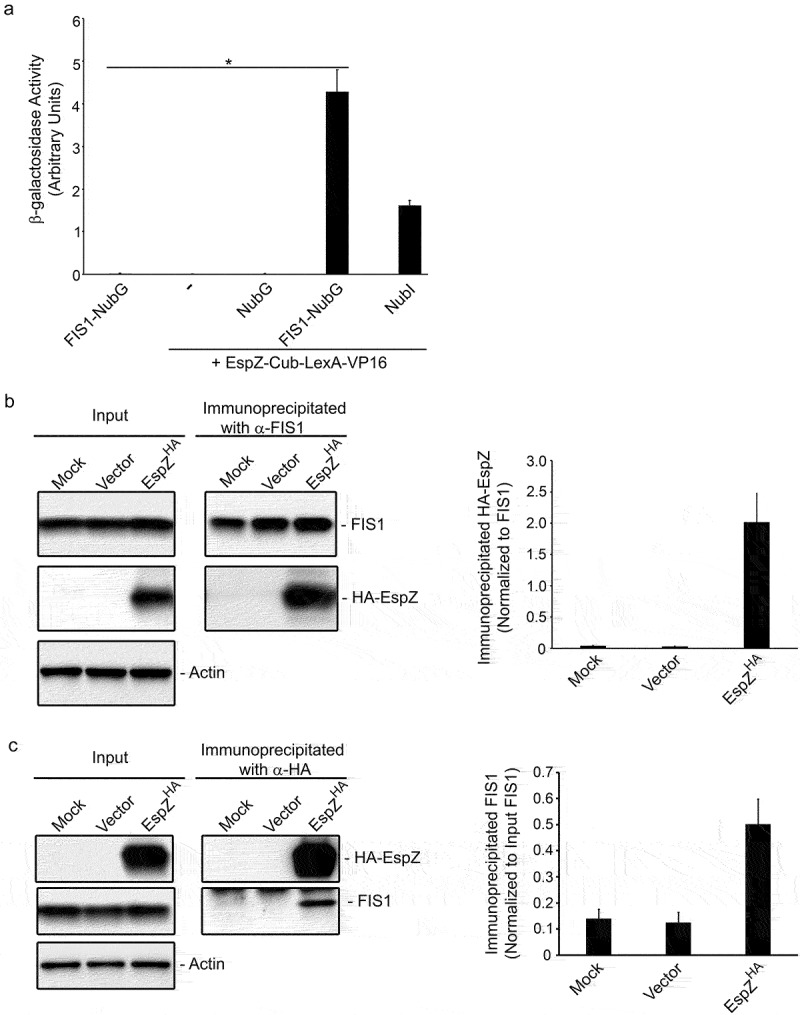
FIS1 interacts with EPEC EspZ. A, β-galactosidase activity of *S. cerevisiae* NMY32 expressing the ‘bait’ EspZ-Cub-LexA-VP16 and NubG (negative control), NubI (positive control), or FIS1-NubG; cells expressing either ‘bait’ (EspZ-Cub-LexA-VP16) alone or ‘prey’ (FIS1-NubG) alone were included as negative controls. Chart shows mean β-galactosidase units in yeast extracts normalized for protein concentration (n = 3) and is representative of three independent experiments. * denotes p_value_ < 0.0002 for NubG-FIS1 + EspZ-Cub-LexA-VP16 compared to FIS1-NubG, EspZ-Cub-LexA-VP16 alone, or NubG + EspZ-Cub-LexA-VP16. B & C, HeLa cells were mock-treated or transiently transfected with a plasmid expressing HA-tagged EspZ (EspZ^HA^) or empty vector control. At 72-hours post-transfection, total protein extracts from mock-treated and transfected cells were immunoprecipitated using α-FIS1 antibodies (b) or α-HA antibodies (c), and extracts and precipitates blotted for FIS1 or HA. Images shown are representative of three independent experiments. B (right panel), chart depicts densitometry analysis of co-immunoprecipitated HA-EspZ normalized against the bait antibody target FIS1. C (right panel), chart depicts co-immunoprecipitated FIS1 normalized against the total FIS1 in extract.

The EPEC secreted protein EspZ localizes to epithelial cell membranes and contributes to virulence likely via its interaction with host proteins.^30,31^ The split-ubiquitin yeast two-hybrid (SUY2H) screen was specifically designed to identify interacting partners of membrane-anchored proteins.^32,33^ In the SUY2H system, interaction between membrane-associated ‘bait’ and ‘prey’ molecules brings the two halves of ubiquitin (Cub and NubG) together. Ubiquitin-specific proteases recognize the reconstituted ubiquitin and cleave at a downstream ‘GGX’ motif to release the transcription factor, LexA-VP16. LexA-VP16 migrates to the nucleus and induces the expression of genes (*his3* and *ade2*) that allow the yeast strain to grow on plates lacking histidine and adenine; positive clones also express β-galactosidase. We used EspZ as a bait in the SUY2H system and screened a HeLa cell library to identify its host–cell interaction partners. This screen identified the mitochondrial fission protein FIS1 as a putative interaction partner of EPEC EspZ under high stringency conditions (growth in <3 days in the presence of 3-amino-1,2,4-triazole, a HIS3 inhibitor). The interaction was verified via pair-wise transformations and quantitative β-galactosidase assays (Figure 1a). Yeast cells expressing both the EspZ ‘bait’ and FIS1 ‘prey’ proteins had robust β-galactosidase activity compared to cells expressing either protein alone. Negative controls, which included cells expressing the EspZ ‘bait’ alone or in combination with NubG, failed to grow on plates lacking histidine and adenine (not shown) and lacked detectable β-galactosidase activity. For a positive control, cells expressing the EspZ ‘bait’ and NubI (which allows for bait-prey-independent ubiquitin reconstitution) were verified to grow on plates lacking histidine and adenine (not shown), and to have β-galactosidase activity.

We then confirmed EspZ-FIS1 interaction in human epithelial cells via co-immunoprecipitation studies. Protein extracts from transfected HeLa cells expressing HA-tagged EspZ (pEspZ^HA^), or control extracts from mock-treated or vector-transfected cells, were immunoprecipitated with α-FIS1 antibody and immunoblotted for HA-tagged EspZ. While FIS1 was precipitated from mock-treated and transfected HeLa cell lysates, a fusion protein corresponding to HA-tagged EspZ was detected only in the immunoprecipitates of EspZ-expressing cells, confirming EspZ-FIS1 interaction (Figure 1b). For additional validation, reciprocal co-immunoprecipitation studies were performed using a α-HA tag antibody. HA-tagged EspZ was detected and immunoprecipitated from EspZ-expressing HeLa cells. While FIS1 was uniformly expressed in mock-, vector- and pEspZ^HA^-transfected cells, FIS1 was only detected in the immunoprecipitates of EspZ-expressing cells, confirming the specificity of EspZ-FIS1 interaction (Figure 1c).

**Figure 2.**
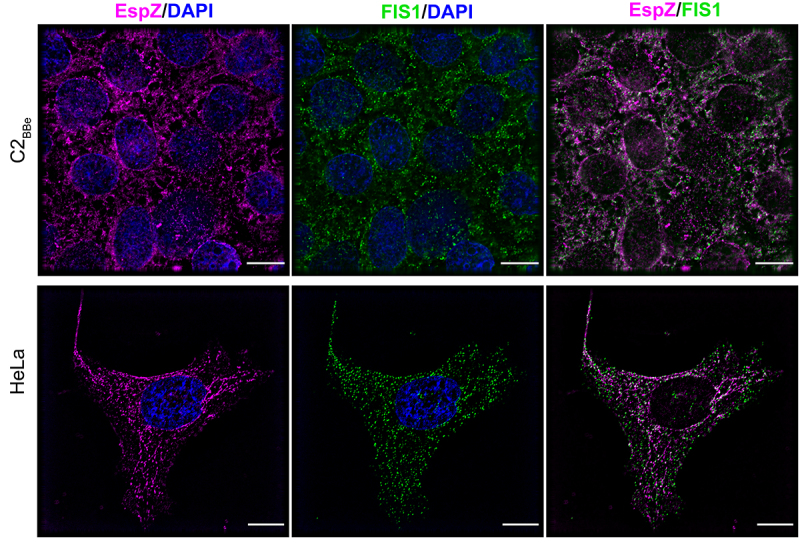
EPEC EspZ colocalizes with mitochondrial fission protein FIS1. C2_BBe_ and HeLa transfected with *espZ-*encoding plasmid (pEspZ^HA^) were fixed and stained for FIS1 (green) and EspZ (magenta). DNA was stained with 4,6- diamidino-2-phenylindole (DAPI; blue). Scale bar: 15 μm (C2_BBe_; top panels) and 10 μm (HeLa; bottom panels). Images shown are representative of >6 images captured from three independent experiments. Overlapping staining of FIS1 and EspZ was confirmed by positive Pearson correlation coefficients of 0.486 ± (C2_BBe_) and 0.795 (HeLa).

To visualize EspZ-FIS1 association in human intestinal epithelial cells, we assessed FIS1 and EspZ distribution in C2_BBe_ cells via immunofluorescence microscopy. Stably transfected EspZ-expressing C2_BBe_ cells and control cells were stained for EspZ, FIS1 and the inner mitochondrial membrane marker COXIV. C2_BBe_ cells have heterogeneously shaped mitochondria with spheroid and tubular structures distributed around the nucleus and extending outward (Supplemental Figure S1a). Consistent with other studies,^34^ and in the absence of EspZ, FIS1 was diffusely distributed within the cytoplasm, with some punctiform FIS1 colocalizing with COXIV-stained mitochondria (Supplemental Figure S1a). Upon ectopic expression, EspZ was visualized as discrete puncta associated with the mitochondria (Supplemental Figure S1b) as shown previously,^30^ and colocalized with FIS1 to the organelle (Figure 2a). Overlapping staining of EspZ to FIS1 was confirmed by a positive Pearson correlation coefficient value (R = 0.486). FIS1 colocalization with EspZ was also observed in human endocervical epithelial HeLa cells (Pearson Correlation Coefficient, R = 0.795), confirming that the interaction is not cell-line specific (Figure 2b). Collectively, these data show that EspZ and FIS1 are targeted to the mitochondria and that they interact with each other in intestinal epithelial cells.

### EPEC infection increases FIS1 abundance in epithelial cells

**Figure 3.**
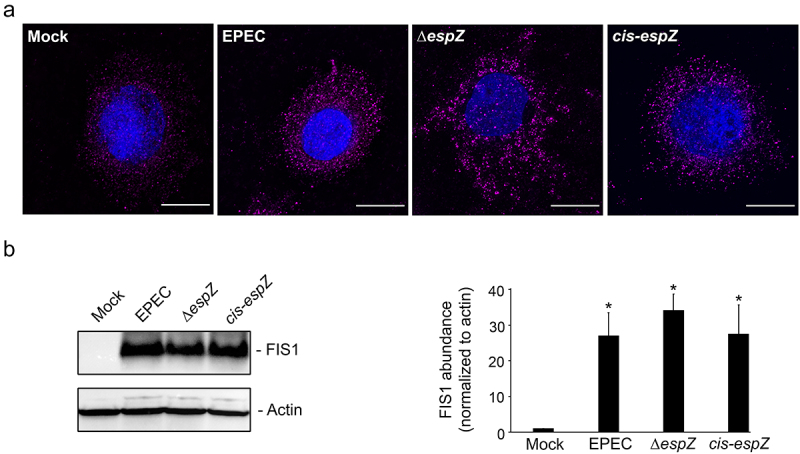
EPEC infection increases FIS1 levels. C2_BBe_ cells were mock-treated or infected with WT EPEC, ∆*espZ*, or *cis-espZ*. A, Immunofluorescence staining of mock-treated or infected C2_BBe_ for FIS1 (magenta); DNA was stained with DAPI (blue). Scale bar: 10 μm. Images shown are representative of >6 images captured from three independent experiments. Exposure time and light transmittance were maintained for all image captures. B, Total protein extracts from mock-treated or infected C2_BBe_ cells were blotted for FIS1. Actin was used as a loading control. Image shown is representative of five independent experiments. The chart depicts densitometry analysis of FIS1 abundance normalized to actin. * denotes p_value_ < 0.0001 for specific sample group compared to mock.

Next, we assessed if EPEC infection influenced FIS1 levels in epithelial cells. C2_BBe_ cells were mock-treated or infected with wild-type EPEC, an isogenic strain with a nonpolar disruption of *espZ* (∆*espZ*), or the corresponding *cis*-complemented mutant strain (*cis-espZ*), and visualized via immunofluorescence for FIS1 alterations. Compared to mock-treated cells, there was a pronounced increase in FIS1 levels in EPEC-infected C2_BBe_ cells; notably, this increase was also evident in Δ*espZ-* and *cis-espZ-*infected host cells (Figure 3a). In immunoblot analyses of infected cells, FIS1 was barely detected in mock-treated cells, but increased over 25-fold in EPEC-, Δ*espZ-* and *cis-espZ-*infected epithelial cells (Figure 3b). Quantitative RT-PCR analyses revealed a 0.704 ± 0.075-fold increase in *fis1/gapdh* transcript levels in EPEC-infected relative to mock-treated C2_BBe_ cells, suggesting that transcriptional upregulation at least partially contributes to increased FIS1 levels. Thus, EPEC infection of intestinal epithelial cells stimulates an increase in FIS1 levels that is independent of EspZ.

### EspZ inhibits mitochondrial fission and mitophagy in EPEC-infected epithelial cells

**Figure 4.**
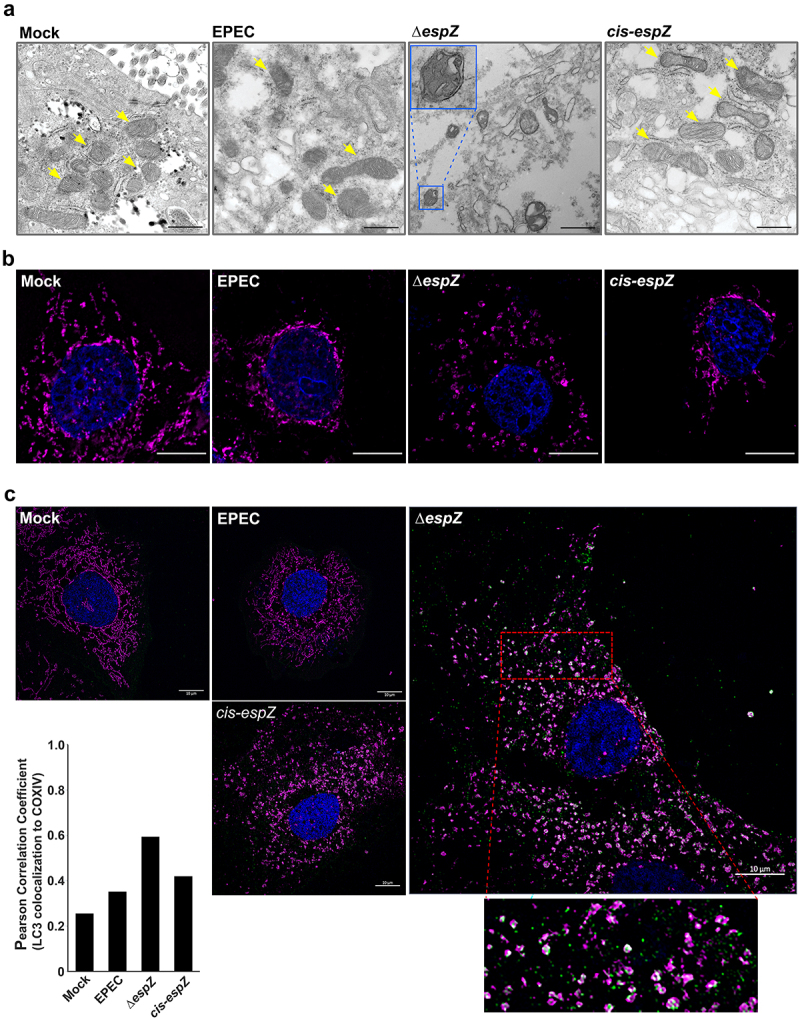
EPEC EspZ inhibits mitochondrial fission and mitophagy. C2_BBe_ cells were mock-treated or infected with WT EPEC, ∆*espZ*, or *cis-espZ*. A, Transmission electron micrographs of mock-treated (left-most panel) or infected C2_BBe_ showing mitochondria (yellow arrows). Inset shows mitochondria enclosed within double membranes, suggestive of mitophagy. Scale bar: 500 nm. Images are representative of >6 fields captured per sample from five independent experiments. B, Immunofluorescence staining of mock-treated or infected C2_BBe_ for COXIV (magenta); DNA was stained with DAPI (blue). **C**, Mock-treated or infected C2_BBe_ were stained for COXIV (magenta), LC3 (green) and DAPI (blue). Scale bar: 10 μm. Inset shows zoomed image of LC3 colocalization with mitochondria. Images shown are representative of >6 images captured from three independent experiments. Chart depicts Pearson correlation coefficients of LC3 colocalized to COXIV.

Mitochondrial position and structure are dynamically regulated, with their shapes ranging from small discrete spheroids to tubular networks that span the entire cell.^35^ The twin processes of fusion and fission regulate mitochondrial shape in response to cellular cues, including stress. Defective mitochondria can be segregated via fission, enclosed in a double membrane, and eliminated by a lysosome-dependent pathway called mitophagy. Increased FIS1 levels can promote both mitochondrial fission and mitophagy.^36^ Therefore, we used transmission electron microscopy (TEM) to assess if EPEC-induced FIS1 upregulation also resulted in mitochondrial morphology changes. Unexpectedly, despite increased FIS1 abundance, there was little evidence of heightened fission or mitophagy in EPEC-infected cells. Indeed, compared to the predominantly spheroid morphology in uninfected C2_BBe_ cells, mitochondria in EPEC-infected cells were more heterogenous, often manifesting as elongated structures (Figure 4a). In contrast, Δ*espZ-*infected cells exhibited a dramatic increase in the number of swollen and rounded mitochondria, with many of these organelles surrounded by double-membranes (Figure 4a; inset), suggestive of mitophagy. Nuclear condensation is also evident in some Δ*espZ-*infected cells, consistent with earlier reports.^37^ Complementation (*cis-espZ*) restored mitochondrial morphology to that seen with WT-infected cells. The mitochondrial size and structural differences were verified via size estimations from the two-dimensional images, and average mitochondrial lengths (from >20 fields for each condition visualized) were as follows: 378 ± 32 nm (Mock-treated); 704 ± 71 nm (EPEC-infected); 461 ± 22 nm (Δ*espZ*-infected) and 711 ± 57 nm (*cis-espZ* infected).

Next, we assessed cellular level changes in mitochondrial positioning and structure during infection. COXIV staining of mock-treated C2_BBe_ cells revealed a uniform mitochondrial network extending from the nucleus to the periphery (Figure 4b). In WT EPEC-infected cells, there was minimal staining in the cytosol, with retraction and clustering of the mitochondrial network around the nucleus. In Δ*espZ-*infected cells, however, there was markedly uneven and dispersed punctate COXIV staining, suggestive of extensive mitochondrial fission. The phenotype reverted to WT morphology in cells infected with the complemented mutant (*cis-espZ)*. Similar mitochondrial phenotypes were observed in EPEC-infected HIEC-6 and HeLa cells, suggesting that these alterations are not cell-line specific (not shown).

During mitophagy, damaged mitochondria are enclosed within a double membrane (as shown in Δ*espZ*-infected cells in Figure 4a) that subsequently fuse with the lysosome.^38^ To verify mitophagy, we assessed localization of the autophagy marker LC3 with COXIV-stained mitochondria. Cells mock-treated or infected with EPEC and *cis-espZ* have low numbers of LC3 associated with the mitochondria, confirmed by low Pearson correlation values (Figure 4c). Compared to mock-treated or EPEC- and *cis-espZ*-infected cells, there was an increase in punctate LC3 staining that co-localized with COXIV-stained mitochondria in Δ*espZ*-infected cells, which was also confirmed by an increased positive Pearson correlation coefficient (Figure 4c).

**Figure 5.**
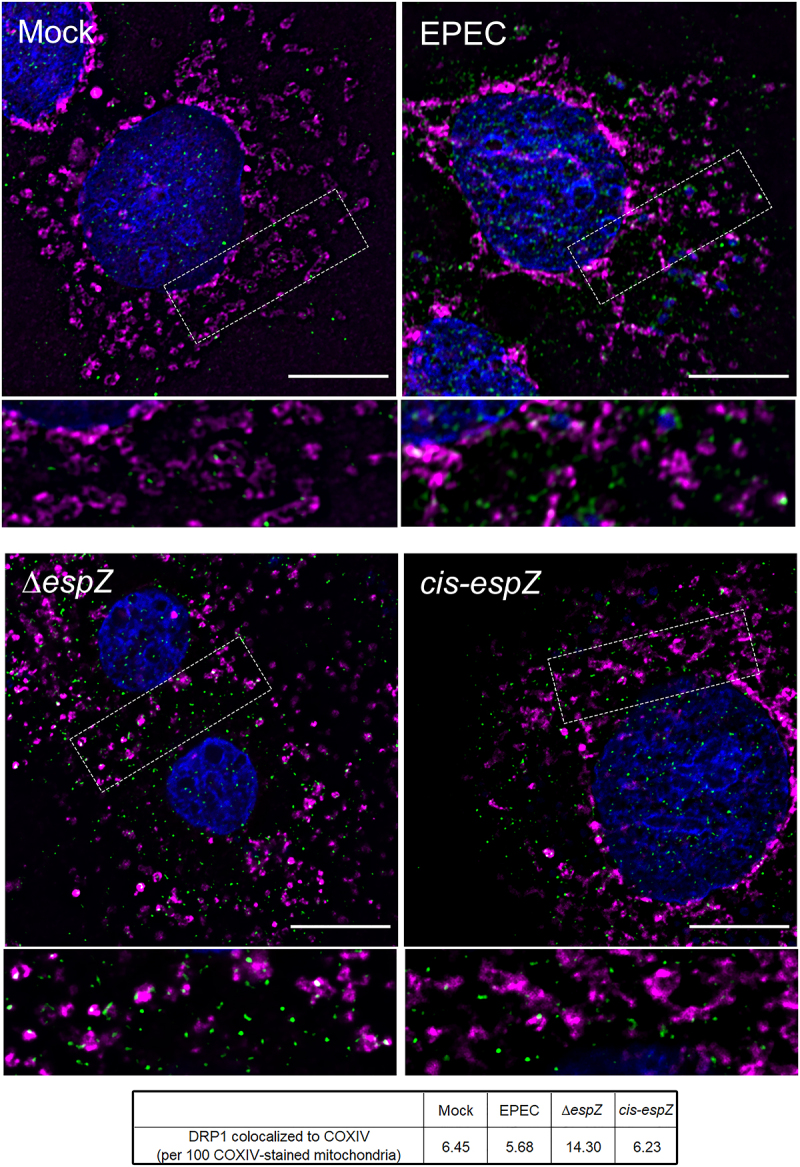
EspZ inhibits DRP1-mediated mitochondrial fission in EPEC-infected enterocytes. C2_BBe_ cells were mock-treated or infected with WT EPEC, ∆*espZ*, or *cis-espZ*, and stained for COXIV (magenta), DRP1 (green), and DAPI (blue). Scale bar: 10 μm. Regions in hatched rectangles were enlarged in lower panels. Images shown are representative of >6 images captured from three independent experiments. Mean number of DRP1 puncta colocalized to the COXIV-stained mitochondria for each sample group is listed in the table. P values for specific sample group compared to mock are shown.

Fission, which precedes mitophagy, relies on recruitment of the GTPase DRP1 (**d**ynamin-**r**elated **p**rotein 1) to the mitochondria by several proteins, including FIS1.^34,35,39–41^ Therefore, we assessed DRP1 localization in EPEC-infected C2_BBe_ cells. Immunofluorescence staining revealed diffuse cytosolic DRP1 staining, with a faint punctate pattern, in mock-treated, EPEC-infected, and *cis-espZ*-infected cells (Figure 5). In contrast, there was abundant mitochondria-associated DRP1 clusters in Δ*espZ-*infected cells. Taken together, and consistent with its localization and interaction, these results suggest that the early-secreted effector EspZ inhibits FIS1/DRP1-dependent mitochondrial fission and mitophagy in EPEC-infected cells, likely by sequestering FIS1.

### EspZ inhibits CCCP-induced, but not LLO-induced, mitochondrial fragmentation

**Figure 6.**
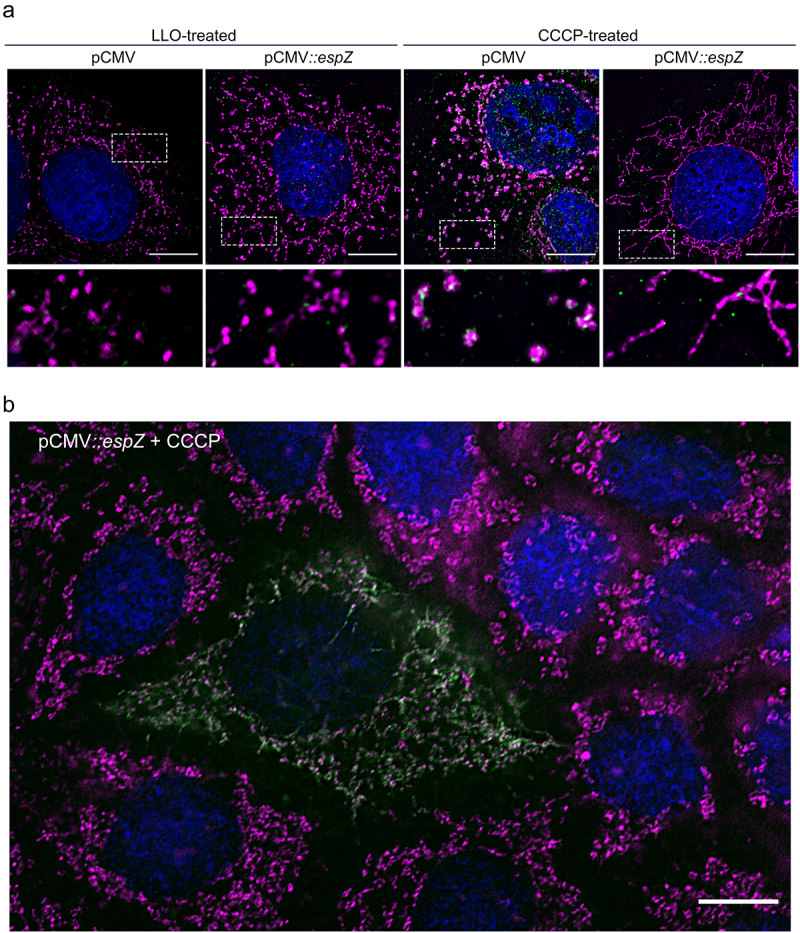
EPEC EspZ inhibits CCCP-induced, but not LLO-induced, mitochondrial fragmentation. (a), Stably transfected C2_BBe_ cells (pCMV-EGFP or pCMV::*espZ*) were treated with carbonyl cyanide m-chlorophenylhydrazone (CCCP) or listeriolysin O (LLO) for 30 minutes and stained for COXIV (magenta), DRP1 (green), and DAPI (blue). Scale bar: 10 μm. Regions in hatched rectangles were enlarged in lower panels. (b), Un-transfected and transiently transfected C2_BBe_ cells (pCMV::*espZ*) were treated with CCCP for 30 minutes and stained for FLAG (green) to visualize FLAG-tagged EspZ, COXIV (magenta) and DAPI (blue). Scale bar: 10 μm. Images shown are representative of >6 images captured from three independent experiments.

Berger et al.^37^ first showed that EspZ acts as a rheostat to regulate translocation of effectors into host epithelial cells. Thus, the EspZ-dependent mitochondrial changes noted above could be direct, or via epistatic control of another effector(s) impact on this organelle. Therefore, we assessed if EspZ, by itself, could protect against mitochondrial fission induced by the uncoupler carbonyl cyanide m-chlorophenyl-hydrazine (CCCP), or by the *Listeria monocytogenes* toxin, listeriolysin O (LLO). Whereas CCCP induces canonical FIS1/DRP1-dependent mitochondrial fission, LLO acts through a poorly understood FIS1/DRP1-independent mechanism.^42–44^ Vector-transfected C2_BBe_ cells treated with either CCCP or LLO displayed punctate COXIV staining, consistent with mitochondrial fission/fragmentation (Figure 6a). DRP1 re-localized to the mitochondria in CCCP-, but not LLO-, treated vector-transfected C2_BBe_ cells, consistent with earlier studies.^43,44^ Stable C2_BBe_ transfectants expressing EspZ, however, were protected against CCCP-induced, but not LLO-induced, mitochondrial fragmentation and perinuclear retraction (Figure 6a). The resistance of EspZ-expressing cells to uncoupler-induced mitochondrial fragmentation was strikingly apparent in transiently transfected C2_BBe_ cells (Figure 6b). In a mixed field of cells, an EspZ-expressing cell resisted CCCP-induced mitochondrial fission, unlike the surrounding cells that did not express EspZ. Collectively, our data confirm that EspZ, independent of any role in regulating effector translocation, directly protects epithelial cells against FIS1/DRP1-dependent mitochondrial fragmentation.

### FIS1 depletion curtails EPEC-induced mitochondrial fission and mitophagy

**Figure 7.**
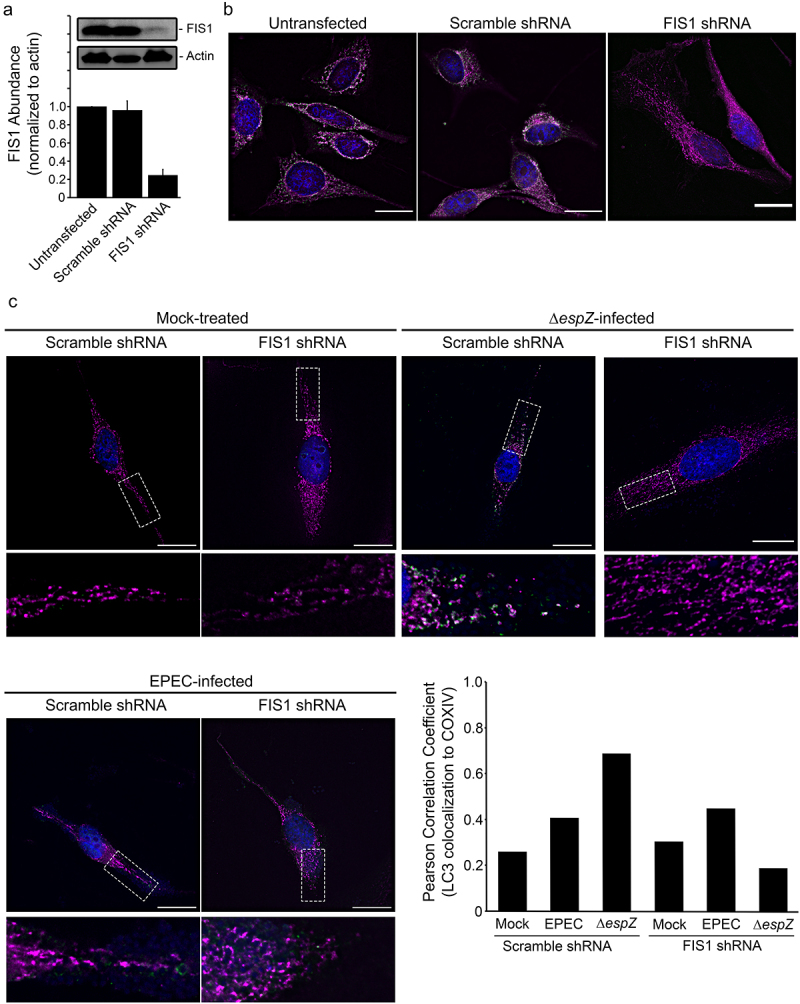
FIS1 depletion protects against EPEC-induced mitophagy. HeLa cells were mock-treated, or transfected with FIS1 shRNA or Scramble shRNA. A, At 7 days post-transfection, total protein extracts from un-transfected and shRNA-transfected HeLa were immunoblotted for FIS1. Actin was blotted as a loading control. Chart depicts densitometry analyses of FIS1 abundance normalized against actin. B, HeLa cells (untransfected or transfected with FIS1 shRNA or Scramble shRNA) were fixed and stained for FIS1 (green), COXIV (magenta) and DAPI (blue). Scale bar: 10 μm. C, HeLa cells transfected with FIS1 shRNA and Scramble shRNA control were mock-treated or infected with EPEC or Δ*espZ*. Cells were fixed, and then stained for LC3 (green), COXIV (magenta) and DAPI (blue). Scale bar: 20 μm. Regions in hatched rectangles were enlarged in lower panels. Images shown are representative of >6 images captured from two independent experiments. Chart depicts Pearson correlation coefficients of LC3 colocalized to COXIV.

To interrogate the specific role of FIS1 in infection-induced mitophagy, we assessed the impact of Δ*espZ* on FIS1-depleted cells. We first verified shRNA-mediated FIS1 down-regulation relative to mock-treated and Scramble shRNA-transfected epithelial cells (~75%; Figure 7a). FIS1 depletion resulted in long mitochondrial extensions projecting out from the nucleus, and increased cell size/volume (Figure 7b), as has been noted previously for primary human fibroblasts, human neuroblastoma M17 cells, and monkey fibroblasts.^40,45–48^ EPEC infection resulted in minimal LC3 recruitment to the mitochondria in both Scramble shRNA- and FIS1 shRNA-transfected cells (Figure 7c). In Scramble shRNA-transfected epithelial cells (and similar to the un-transfected cells; not shown), Δ*espZ* infection caused perinuclear mitochondrial retraction, and increased abundance of small spheroid mitochondria that were associated with LC3, which was confirmed by an increased positive Pearson correlation coefficient (Figure 7c), suggestive of mitophagy. In Δ*espZ* infected FIS1-depleted cells, however, mitochondrial morphology more closely resembled mock-treated cells, and absence of LC3 recruitment; the Pearson correlation coefficient value for LC3/COXIV colocalization was lower in Δ*espZ* infection of FIS1-depleted cells compared to infected Scramble shRNA-transfected cells (Figure 7c). Further, while Δ*espZ* infection increased DRP1 localization to the mitochondria in Scramble shRNA-transfected cells, this was not observed in FIS1-depleted cells (Supplemental Figure S2). Taken together, our data suggest that EPEC infection markedly elevates FIS1 levels in epithelial cells, but EspZ effectively blocks FIS1/DRP1-dependent mitochondrial fission and mitophagy.

### FIS1 depletion curtails EPEC-induced host cell death

**Figure 8.**
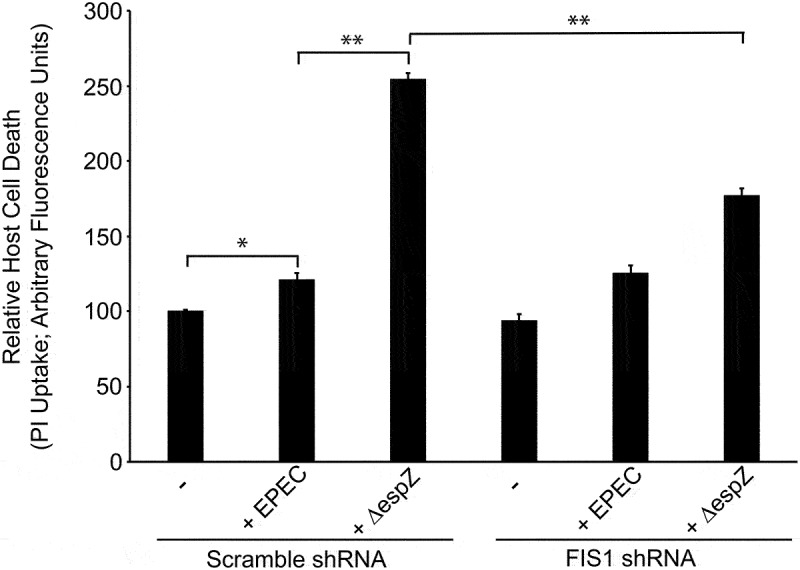
FIS1 depletion curtails EPEC-induced host cell death. HeLa cells were transfected with FIS1 shRNA or Scramble shRNA. At 7 days post-transfection, cells were infected with Δ*espZ* at a multiplicity of infection of 100. At 1-hour post-infection, unattached bacteria were removed. Host cell death was then measured via propidium iodide (PI) uptake at 3 hours. Chart shown is representative of 3 independent experiments (n = 5 per sample group). * denotes p_value_ <0.0001 and ** denotes p_value_ < 0.05 for specific group comparisons in brackets.

We and others have previously demonstrated that EspZ limits and delays the death of EPEC-infected intestinal epithelial cells.^29–31^ To interrogate whether FIS1 interaction contributes to the cytoprotective effect of EspZ, we assessed EPEC-mediated host cell death in FIS1-depleted cells. In Scramble shRNA-transfected cells, EPEC induced a modest increase in host cell death relative to mock-treated cells, and this was significantly increased following Δ*espZ* infection (Figure 8), consistent with earlier studies.^29–31^ While FIS1 depletion did not impact EPEC-induced host cell death, FIS1 depletion significantly curtailed Δ*espZ*-induced cell death, supporting a role for EspZ-FIS1 interaction in promoting the survival of infected cells (Figure 8).

### EspH upregulates FIS1 and curtails EPEC-induced mitochondrial fusion

**Figure 9.**
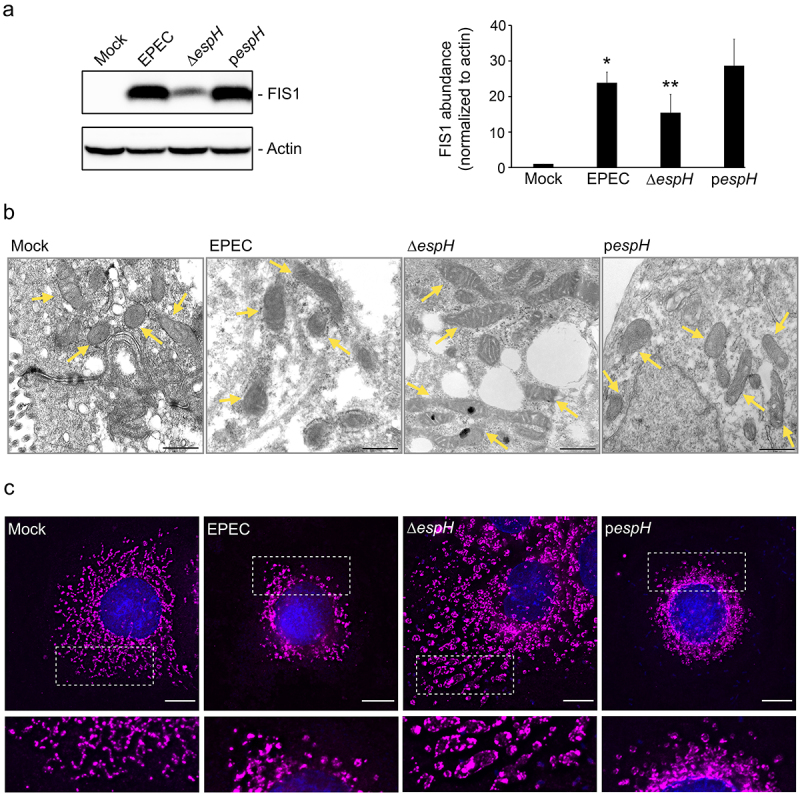
EspH increases FIS1 levels and promotes mitochondrial fission. C2_BBe_ cells were mock-treated or infected with WT EPEC, ∆*espH*, or p*espH*. A, Total protein extracts from mock-treated or infected C2_BBe_ cells were blotted for FIS1. Actin was blotted as a loading control. Image shown is representative of four independent replicates. Chart depicts densitometry analyses of FIS1 abundance normalized against actin. * denotes p_value_ <0.0001 and ** denotes p_value_ < 0.05 for specific group comparisons in brackets. B, Transmission electron micrographs of mock-treated (left-most panel) or infected C2_BBe_ showing mitochondria (yellow arrows). Scale bar: 500 nm. Images are representative of >6 fields captured per sample from two independent experiments. C, Mock-treated or infected C2_BBe_ were stained for COXIV (magenta) and DAPI (blue). Scale bar: 10 μm. Regions in hatched rectangles were enlarged in lower panels. Images shown are representative of >6 images captured from three independent experiments.

We next sought to identify the EPEC factor(s) contributing to increased FIS1 abundance in infected cells. Initial infection studies with a T3SS-deficient strain suggested that this was primarily driven by one or more secreted effectors. We hypothesized that the most plausible candidates would be the mitochondria-targeted effectors EspF and Map, or the cytotoxic effector EspH (which inhibits Rho GTPase and causes cytoskeletal perturbations). Infection of C2_BBe_ cells with the corresponding mutants showed that, like WT EPEC, Δ*map* and Δ*espF* upregulated FIS1 levels relative to mock-treated cells. In contrast, FIS1 abundance was markedly lower in Δ*espH-*infected cells (Supplemental Figure S3). C2_BBe_ cells infected with WT EPEC and the complemented (p*espH*) strain had greater abundance of FIS1 relative to mock-treated and Δ*espH-* infected cells (Figure 9a). Thus, EspH is the primary driver of EPEC-induced increase in epithelial cell FIS1 abundance.

We then performed TEM and immunofluorescence studies to assess the impact of EspH on mitochondrial morphology. While WT EPEC infection induced a modest increase in mitochondrial size relative to mock-treated cells (as in Figure 4a), Δ*espH* infection caused a dramatic increase in mitochondrial length, and this was reversed by complementation with plasmid-encoded *espH* (p*espH;* Figure 9b). Specifically, mitochondria in Δ*espH*-infected cells had morphology reminiscent of profound fusion with average organelle length in some cases exceeding 1429 ± 161 nm (in contrast to average mitochondrial length of 486 ± 51 nm for mock-treated, 687 ± 62 nm for EPEC-infected, and 691 ± 46 nm for p*espH*-infected cells). The mitochondrial size changes in infected cells were confirmed via super-resolution microscopy imaging (Supplemental Figure S4). Consistent with electron microscopy visualizations, COXIV staining revealed elongated mitochondria in Δ*espH-*infected cells, but not in WT- or p*espH*-infected cells (Figure 9c), implicating a role for EspH in promoting mitochondrial fission. Collectively, our data illustrate the opposing actions of EspZ and EspH on FIS1-dependent mitochondrial fragmentation and mitophagy in EPEC-infected intestinal epithelial cells.

Membrane potential (ΔΨm) assays revealed that EspZ and EspH had contrasting effects on mitochondrial function, consistent with their impacts on the organelle structure (Supplemental Figure S5a). Relative to mock-treated cells, EPEC decreased ΔΨm; Δ*espH* failed to induce mitochondrial membrane potential loss. In contrast, Δ*espZ*-infected cells had significantly greater ΔΨm disruption relative to WT-infected cells, suggesting that EspZ prevents mitochondrial membrane depolarization, consistent with a previous report.^30^ We therefore assessed whether EspZ, by itself, can protect against CCCP-induced ΔΨm loss (Supplemental Figure S5b). The uncoupler CCCP disrupted the ΔΨm of untransfected and vector-transfected C2_BBe_, while EspZ-expressing cells resisted CCCP-induced ΔΨm loss.

Collectively, our data show that EPEC stimulates mitochondrial fragmentation via EspH-dependent increase in FIS1 levels, but this is suppressed or delayed via EspZ sequestration of FIS1. Correspondingly, EspZ preserves mitochondrial membrane potential, while EspH causes a loss in membrane potential.

## Discussion

EspZ is critical for the virulence of A/E pathogens REPEC (rabbit EPEC) and *Citrobacter rodentium* in rabbits and mice, respectively; isogenic *espZ* deletion mutants of *C. rodentium* and REPEC were severely attenuated for intestinal colonization and virulence.^4,8,49^ Among typical EPEC isolates (that elaborate the bundle-forming pilus), the presence of *espZ* is significantly correlated with symptomatic clinical outcomes in young children.^50^ We and others previously demonstrated that EspZ curtails the death of EPEC-infected intestinal epithelial cells.^4,29,31,37^ Two mutually non-exclusive mechanisms have been proposed to explain the cytoprotective effect of EspZ: (1) EspZ acts as a rheostat to limit the translocation of proteins, including cytotoxic effectors, into host cells^37^ and (2) EspZ, via interaction with host proteins, directly modulates intestinal epithelial cell death pathways.^4,29–31^ Consistent with the latter mechanism, our current data suggest that EspZ localizes to the mitochondria, interacts with the fission protein FIS1, and limits DRP1-dependent fission and mitophagy. Previously, Shames et al.^30^ identified the inner mitochondrial membrane protein TIM17b as an interactor of EspZ. EPEC infection of TIM17b-depleted cells resulted in increased cytotoxicity. The domains of EspZ involved in host cell interactions remain undefined, and it is presently not clear how it may interact with an outer membrane protein-like FIS1, as well as the inner membrane-localized TIM17b. To date, there are no reports implicating a role for TIM17b in mitochondrial morphology changes.

Our studies also uncover a novel role for EspH in increasing FIS1 abundance in host cells and in limiting mitochondrial fusion. EspH, a multi-functional effector inhibits Rho GTPases, has pleiotropic impacts on epithelial cells including cytoskeletal alteration, caspase activation, and induction of cell death.^22–25 51^ EspH inhibits Rho GTPases possibly via two mechanisms: (1) by its binding to the Dbl-homology and the adjacent pleckstrin-homology (DH-PH) domain, and sequestration, of RhoGEFs,^22,25^ and/or (2) its stimulation of RhoGAP activity.^51^ EspH also interacts with CD81 tetraspanin microdomains and suppresses mitogen-activated protein kinase/extracellular signal-related kinase (Erk) activity.^52^ We recently demonstrated a key role for EspH in altering the cytokeratin network and perturbing desmosomal junctions.^24^ EspH is not required for initial colonization, but is essential for bacterial persistence in the intestine, and for inducing robust disease symptoms.^24,53^ An EspH-deficient EHEC strain (Δ*espH*) had reduced colonization throughout the intestine and induced only mild/moderate diarrhea, compared to the severe diarrhea in WT-infected rabbits.^53^ EspH does not harbor a canonical mitochondrial targeting sequence, and the mechanism(s) by which it increases FIS1 abundance is an area of active investigation. Further, the potential relationship between its various functions and their contribution to virulence needs further exploration.

Cells infected with an isogenic Δ*espH* strain had moderately increased FIS1 levels relative to mock-treated cells, but considerably less than that induced by WT EPEC infection. This suggests that other EPEC factors could play a role in increasing FIS1 levels in host cells. Δ*map* and ΔespF strains were comparable to WT for FIS1 upregulation, suggesting a role for alternate factors, possibly other effector proteins. A recent study demonstrated Map-dependent increase in DRP1 expression, and decrease in MFN1/MFN2 expression, in bovine mammary epithelial cells.^54^ Correspondingly, Map promoted DRP1 dependent mitochondrial fission and apoptosis; it remains to be determined if Map and EspH coordinate to increase mitochondrial fission. It is curious that an isogenic Δ*espH* strain (expected to express and secrete Map and EspF) not only fails to display fragmentation but, rather, exhibits hyper-fused mitochondria.

FIS1 regulates mitochondrial morphology via distinct mechanisms in mammalian cells.^55,56^ Fission relies on recruitment of the large, highly regulated GTPase, dynamin-related protein 1 (DRP1) to the mitochondria; DRP1 multimerization on the organelle surface leads to constriction and separation.^57^ FIS1, as well as MFF, MiD49 and MiD51, can recruit DRP1 to the mitochondria.^34,39–41^ Alternately, FIS1 can directly interact with MFN1 and MFN2 to inhibit mitochondrial fusion.^55^ The robust induction of mitochondrial fragmentation and mitophagy in Δ*espZ-*infected cells, and of hyperfused mitochondria in Δ*espH-*infected cells are consistent with the recently proposed dual role for FIS1 in promoting DRP1-dependent fission and inhibiting MFN-dependent fusion.^55^

Prior studies suggest nuanced regulation of mitochondrial morphology and function by A/E pathogens.^16,18,20,21,27,30,58,59^ Besides directly interacting with host proteins to alter mitochondrial functions, EspZ’s rheostat function could limit the entry of effectors that damage epithelial cell mitochondria. The translocation of the effector Map into mitochondria depends on maintenance of an intact membrane potential.^18^ EspZ, by preserving mitochondrial membrane potential, could thus facilitate Map import into the organelle and mediate downstream effects. Proteomic studies of colonic epithelial cells from mice infected with *C. rodentium* for 8 days suggested a downregulation of all TCA cycle enzymes and most of the proteins involved in the electron transfer chain.^59^ The consequent switch of infected cells to reliance on aerobic glycolysis, and resulting increased oxygenation at mucosal surfaces, was proposed to promote *C. rodentium* colonization. This may represent an infection stage prior to overt and extensive mitophagy and host cell death, possibly induced by EspH and other effectors.

It is increasingly evident that pathogens manipulate host mitochondrial structure and function as a virulence strategy.^60–64^ Infection of various human cell types with *Chlamydia trachomatis* increased mitochondrial fusion, possibly via phosphorylation-dependent DRP1 inhibition, and increased respiratory activity and ATP production.^65,66^ DRP1 overexpression increased mitochondrial fragmentation and prevented establishment of *Chlamydia* infection.^65^ There are more examples of pathogens that promote mitochondrial fragmentation, and the mechanistic details have been defined in many instances.^61–64^ The *Vibrio cholerae* protein VopE decreased MFN1-induced mitochondrial fusion and reduced perinuclear mitochondrial clustering by inhibiting the activity of the mitochondrial Rho GTPase Miro1; VopE-targeted mitochondrial changes dampened innate immune signaling, and promoted survival in the infected host.^67^ *Shigella flexneri* caused DRP1-dependent mitochondrial fragmentation and host cell death, and DRP1 depletion or inhibition resulted in reduced *Shigella* plaque formation.^68^ Similarly, the *Legionella pneumophila* secreted effector MitF promoted DRP1-dependent mitochondrial fission, and mitochondrial respiration was halted in infected cells.^69^ In contrast, the listerial protein listeriolysin O triggered mitochondrial fission via a non-canonical DRP1-independent pathway,^43^ and disrupted membrane potential, and ATP production. Alteration of either fission or fusion affected the efficiency of *Listeria monocytogenes* infection.^44^

Collectively, our data and the published literature show that EPEC, an extracellular pathogen, orchestrates intestinal epithelial mitochondrial alterations, and this could underlie the critical requirement of effector proteins like EspZ and EspH for optimal virulence of A/E pathogens.

## Materials and methods

### Split-ubiquitin yeast two-hybrid (SUY2H) screen

The bait plasmid (EspZ-Cub-LexA-VP16) was constructed by amplifying wildtype EPEC *espZ* and cloning into pBT3-STE (Table 1). EspZ-Cub-LexA-VP16 expresses EspZ fused to the C-terminal half of ubiquitin (Cub) and the artificial transcription factor LexA-VP16. Prey plasmids were derived from a HeLa cell library with cDNA inserts cloned in pPR3-N plasmid (Table 1). Prey plasmids expressed proteins fused to the mutated N-terminal half of ubiquitin. SUY2H screen was performed by Dual Systems Biotech AG (Grabenstrasse, Switzerland; now under Hybrigenics Services, Evry, France) using EspZ-Cub-LexA-VP16 and HeLa prey library.

**Table 1.** List of plasmids.

Plasmid	Alternate Name	Description/Relevant Genotype or Phenotype	Antibiotic Resistance	Reference or Source
pBT3-STE		Empty Y2H bait vector		Hybrigenics Services (Evry, Franch); formerly sold by DualSystems Biotech (Grabenstrasse, Switzerland)
EspZ-Cub-LexA-VP16		Y2H bait plasmid		
pPR3-N		Empty Y2H prey vector		
FIS1-NubG		Y2H prey plasmid expressing FIS1		
NubI		Y2H Positive prey control		
NubG		Y2H Negative prey control		
pCIP-TTL-HA	pSR4	Lentiviral expression control plasmid based on the pCIP-TTL-HA backbone	Amp^r^, Pur^r^	^4^
pEspZ^HA^	pSR3	Plasmid for transient expression of EPEC *espZ-*HA in eukaryotic cells; EPEC *espZ* allele cloned into pSR4; alternatively called pSR3 in previous study	Amp^r^, Pur^r^	^4^
pCIP-TTL-FLAG		Lentiviral expression control plasmid based on the pCIP-TTL-FLAG backbone	Amp^r^, Pur^r^	This study
pSUPERIOR.retro.neo+gfp	Scramble shRNA	pSuperior RNAi vector system with Scramble shRNA for negative control cells	Amp^r^, Neo^r^	OligoEngine (Seattle, WA)
pSUPERIOR.retro.neo+gfp-Fis1	FIS1 shRNA	pSuperior RNAi vector system for FIS1 shRNA expression in mammalian cells	Amp^r^, Neo^r^	OligoEngine

Note: Amp^r^, Cm^r^, Pur^r^, and Neo^r^ denote resistance to ampicillin, chloramphenicol, puromycin, and neomycin respectively.

### β-galactosidase activity assay

Yeast cells were transformed with the SUY2H empty bait vector (pBT3-STE), bait plasmid (EspZ-Cub-LexA-VP16), empty prey vector (pPR3-N), FIS1 prey plasmid (FIS1-NubG), positive prey protein (NubI) or negative control protein (NubG) (Table 1). β-galactosidase activity is the readout for positive bait and prey interactions. Yeast cell β-galactosidase activity was monitored by assessing the hydrolysis of o-nitrophenyl β-D-galactopyranoside (ONPG) to the yellow product o-nitrophenol via absorbance measurements at 420 nm (Abs_420nm_). In brief, proteins extracted from yeast cells grown overnight were quantitated using a BCA assay (Bio-Rad, Hercules, CA). Yeast protein extracts (0.1 mL) were incubated with ONPG, Z-buffer, and β-mercaptoethanol at 30°C. Following development of the yellow color, sodium bicarbonate (1 mol/L) was added, elapsed reaction time recorded in minutes, tubes centrifuged at 16,000xg for 10 minutes, and supernatants transferred to clean cuvettes. Abs_420nm_ of the samples relative to the blank was then recorded. β-galactosidase units were calculated using the formula:

[Abs_420nm_ × 1.7]/[0.0045 × protein concentration × extract volume × time]

Where protein concentration was in mg/mL as determined by the BCA; extract volume = 0.1 mL; time = elapsed reaction time recorded in minutes

### Bacterial strains and generation of mutants

Plasmids, primers, and bacterial strains used in this study are listed in Tables 1, 2 and 3, respectively. A nalidixic acid-resistant derivative of EPEC O127:H6 strain E2348/69 was used in this study. The isogenic, nonpolar *espZ* deletion in EPEC (Δ*espZ*) was generated using a SacB-based counter-selection method.^71,72^ The single-copy *cis*-complemented strain (*cis-espZ*) was constructed via Tn7 transposition.^29,73^ The Δ*espH* and corresponding complemented strain (p*espH*) were previously described.^24^ Attachment assays were performed to confirm comparable host cell attachment to C2BBe cells by EPEC and mutants (Table 3).

**Table 2.** List of primers.

Primer	Sequence (5’-3’)
SR48	GATCATCTAGAATGGAAGCAGCAAATTTAAGCCCTT
SR49	AAAATCCGGAGGCATATTTCATCGCTAATCCGC

**Table 3.** List of strains.

Strain Name	Alternate Name	Description/Relevant Genotype or Phenotype	Antibiotic Resistance	Citation	Percent Host Cell Attachment to C2_BBe_
EPEC	VK003	Nalidixic acid-resistant derivative of EPEC E2348/69 strain (serotype O127:H6) isolated from an infant during an outbreak in Taunton, United Kingdom in 1969; E2348/69	Nal^r^	^70,74^	6.02 ± 0.95
∆*espZ*	MK41	EPEC derivative MK41 with a nonpolar espZ deletion; E2348/69 Nal^r^ Km^r^::*espZ::aphA*-3	Nal^r^, Kan^r^	^71^	7.87 ± 1.4
*cis-espZ*	VK572	∆*espZ* complemented a single copy of wild-type EPEC *espZ*, with its native promoter inserted downstream of *glmS*	Nal^r^, Amp^r^, Kan^r^	^29^	5.17 ± 0.73
∆*espH*	SE874	EPEC derivative with a nonpolar disruption of *espH*; E2348/69 Nal^r^ Km^r^::*espH::aphA*	Nal^r^, Kan^r^	^24^	7.20 ± 1.35
p*espH*	SE874/C	∆*espH* complemented with *espH* cloned in pTrcHis2TOPO-TA; plasmid was designated as pJLR1 in previous study	Nal^r^, Amp^r^, Kan^r^	^24^	8.24 ± 1.24

Note: Amp^r^, Kan^r^, and Nal^r^, denote resistance to ampicillin, kanamycin, and nalidixic acid, respectively.

### Cell lines, propagation, and transfections

The human intestinal epithelial C2_BBe_ cell line, a brush-border-expressing Caco-2 subclone, was cultured as previously reported.^75,76^ The human endocervical epithelial HeLa cell line was cultured in Dulbecco’s Modified Eagle Medium (DMEM; Thermo Fisher Scientific, Waltham, MA) containing 10% FBS (Atlanta Biologicals, Lawrenceville, GA). All cell lines were grown at 37°C in a 5% CO_2_ atmosphere.

To generate plasmids for transient expression of wild-type EPEC EspZ in eukaryotic cells, *espZ* was amplified using primers SR48 and SR49, digested with *Xba*I and *Bsp*EI, and cloned into a similarly digested pCIP-TTL-HA vector, in frame with a C-terminal HA tag, and upstream of the internal ribosome entry site (IRES) sequence to generate pEspZ^HA^ (Table 1). pEspZ^HA^ and the lentiviral expression control plasmid pSR4 were described previously.^4^ For transient transfection studies, HeLa cells grown to 60% confluency were transfected with vector control (pSR4) or plasmid harboring EspZ (pEspZ^HA^) using the JetPRIME transfection reagent (Polyplus Transfections, Illkirch-Graffenstaden, France) according to the manufacturer’s protocol. Cells were used for co-immunoprecipitation studies at 72 hours post-transfection.

To generate stable transfectants expressing green fluorescent protein (GFP) alone or EspZ and GFP, C2_BBe_ cells were transfected with the previously described pCMV-EGFP vector or pCMV::*espZ*.^29^ C2_Bbe_ cells were trypsinized and resuspended in Opti-MEM I reduced serum medium (Thermofisher Scientific). C2_BBe_ cells (10^6^) were electroporated with 30 μg of plasmid DNA (260 V, 850 μF, and 720 Ω) (Bio-Rad Gene Pulser X Cell). Transfected cells were repeatedly sorted for GFP expression by using a BD FACS Aria III cell sorter (Becton Dickinson, San Jose, CA), until >90% of the cells were consistently expressing GFP.

### Infection of epithelial cell lines

Epithelial cells were incubated in serum-free DMEM 3 hours prior to infection. Overnight bacterial cultures in Luria-Bertani broth with appropriate antibiotics were diluted in DMEM and grown to an optical density (OD_600_) of 0.4. C2_BBe_ cells were mock-treated or infected with WT EPEC, ∆*espZ, cis-espZ*, Δ*espH*, or p*espH* at a multiplicity of infection of 100 for 3 hours.

### shRNA knockdown

Hela cells were transiently transfected with FIS1 shRNA or Scramble shRNA. At 72 hours post-transfection, cells were trypsinized and sorted for GFP expression using a BD FACS Aria III cell sorter (Becton Dickinson) and collected in DMEM containing 10% FBS supplemented with 1X gentamycin (Millipore Sigma, St. Louis, MO) and 1X antibiotic-antimycotic (Atlanta Biologicals). GFP-positive cells were then seeded on coverslips for immunofluorescence studies and 96-well microplates for propidium iodide uptake assays. FIS1 knockdown was confirmed via immunoblotting using α-FIS1 as the primary antibody (Santa Cruz Biotechnology, Dallas, TX).

### Co-immunoprecipitation studies

Co-immunoprecipitation studies were performed using transiently transfected HeLa cells expressing HA-tagged EspZ. At 72-hours post-transfection, cells were harvested via centrifugation, and proteins were extracted using IP Lysis/Wash Buffer Supplemented with 1% CHAPs (Thermo Fisher Scientific).^30,37^ Immunoprecipitation was performed on 2 mg protein extracts using α-FIS1 antibody (Santa Cruz Biotechnology), α-HA antibody (Millipore Sigma) and Pierce Crosslink IP Kit (Thermo Fisher Scientific) following manufacturer’s protocol. Immunoprecipitated samples were resolved via a 4–20% SDS-PAGE. The presence of HA-tagged EspZ and FIS1 was confirmed by western blot using α-HA and α-FIS1 antisera (Millipore Sigma).

### Protein extraction

Cultured epithelial cells were harvested and pelleted by centrifugation at 400 g for 7 minutes. For co-immunoprecipitation studies, cell pellets were resuspended in extraction buffer (Thermo Fisher Scientific IP Lysis Buffer supplemented with 1% CHAPS) and sonicated to facilitate cell lysis. Samples were centrifuged to collect the soluble protein fraction. Total protein samples from infection experiments were prepared using a urea-based extraction buffer (7 M urea, 2 M thiolurea, 100 mM dithiotreitol, and 4% CHAPS). Densitometry analyses were performed using ImageJ Version 1.53e.^77^

### Immunoblot experiments

Protein extracts (30–50 μg) were separated via 4–20% sodium dodecyl sulfate-polyacrylamide gel electrophoresis and transferred to 0.2-μm polyvinylidene fluoride membranes. Blots were blocked with 5% nonfat milk in Tris-buffered saline containing 0.1% Tween 20 and incubated with α-EspZ (Alpha Diagnostic International, San Antonio, TX), α-FIS1 (Abcam, Waltham, MA), α-HA or α-actin anti-sera (Millipore Sigma). Horseradish-peroxidase–conjugated goat anti-rabbit and anti-mouse antisera (Millipore Sigma) were used as secondary antisera. Membranes were developed with SuperSignal West Femto Chemiluminescent Substrate (Thermo Fisher Scientific).

### Immunofluorescence microscopy

Cultured HeLa or C2_BBe_ were grown on poly-L-lysine–coated coverslips. Cultured monolayers were fixed in a 1:1 mixture of methanol and acetone for 20 minutes at −20°C, air-dried, rehydrated in PBS for 5 minutes, permeabilized with 0.2% Triton X-100 (Millipore Sigma) in PBS for 15 minutes, and blocked with 5% IgG-free bovine serum albumin (BSA) in PBS for 1 hour. Samples were incubated with fluor-conjugated antibodies for COXIV, FIS1, DRP1, TOM20 or LC3 primary antisera (Abcam) diluted in BSA blocking solution with 0.05% sodium azide overnight at 4 C, and then washed 3 times with 5% IgG-free BSA in PBS. Samples were washed with PBS, stained with 4,6-diamidino-2-phenylindole (DAPI), and mounted in ProLong Diamond Antifade reagent (Thermo Fisher Scientific). Images were captured on a DeltaVision Elite Deconvolution Microscope (GE Healthcare, Pittsburgh, PA) equipped with an Olympus 60x/1.42 objective or an Olympus 100x/1.4 objective with 1.59x auxiliary magnification, Zeiss ELYRA S1 (SR-SIM) Super Resolution Microscope equipped with Plan-Apochromat 63x/1.40 Oil objective and using immersion oil with refractive index, n = 1.516 (Sigma-Aldrich). Fields of infected cells captured were verified to have comparable bacterial attachment by viewing DAPI-stained microcolonies.

### Transmission electron microscopy

Polarized IECs grown on 0.33-cm^2^ collagen-coated Transwells (Thermo Fisher Scientific) were apically infected with EPEC strains cultured in DMEM. IECs were fixed in Karnovsky’s Fixative (Electron Microscopy Sciences, Hatfield, PA) overnight at 4°C, neutralized with 125 mmol/L glycine in PBS, postfixed in 1% osmium tetroxide, and sequentially dehydrated with 15%, 30%, 50%, 70%, 90%, and 100% ethanol. Samples then were infiltrated with Spurr’s Resin (Electron Microscopy Sciences). Ultrathin sections were contrasted with 2% uranyl acetate, followed by Reynold’s lead citrate, and visualized with an FEI Tecnai Spirit transmission electron microscope (FEI, Hillsboro, OR).

### Tetramethylrhodamine, ethyl ester (TMRE) membrane potential (ΔΨm) assays

C2_BBe_ cells (5000 cells/well) were seeded to 96-well black-walled, clear bottom microplates and grown to confluency. Cells were mock-treated or infected as described above. Unattached bacteria were carefully removed at 1-hour post-infection. At 2.5-hours post-infection, Mitochondrial Membrane Potential Assay Kit II TMRE labeling solution (Cell Signaling Technology, Inc., Danvers, MA) was added to a final concentration of 200 nM. After 30 minutes post-TMRE addition (coinciding with 3 hours of infection), culture medium was removed, and cells were washed three times with PBS. TMRE fluorescence readings were taken using a Synergy HT microplate reader equipped with a 530/25 nm excitation and 590/20 nm emission filters (BioTek Instruments, Winooski, VT). For TMRE membrane potential assays performed on CCCP-treated cells, untransfected and stably transfected C2_BBe_ cells (5000 cells/well) described above were similarly seeded to 96-well black-walled, clear bottom microplates and grown to confluency. Cells were incubated in fresh culture medium containing 200 nM TMRE for 30 minutes. After washing cells three times with PBS, initial TMRE fluorescence readings were taken. Cells were incubated with culture medium containing 10 μM CCCP for 10 minutes and then washed three times with PBS, and final TMRE fluorescence readings were taken.

### Propidium iodide uptake assay

Hela cells were transiently transfected with FIS1 shRNA or Scramble shRNA and FACS-sorted as described above. At 72 hours post-transfection, sorted cells (5000 cells/well) were seeded to 96-well black-walled, clear bottom microplates. Seven days after initial transfection, cells were infected as mentioned above. Unattached bacteria were carefully removed at 1-hour post-infection. Fresh medium containing 1 μg/mL of propidium iodide (PI; Abcam, Cambridge, MA) was added to the cells. PI uptake was monitored for 8 hours, and fluorescence readings taken at 30-minute intervals using a microplate reader (Synergy HT; BioTek Instruments, Winooski, VT) equipped with a 530/25 nm excitation and 620/40 nm emission filters. Epithelial cells killed with 70% methanol were used as controls to estimate maximum PI uptake.

### Statistical analysis

All in vitro experiments were performed with a minimum of two independent experiments and ≥ three biological replicates, unless indicated otherwise. Charts depict mean values, and error bars represent standard error of the mean. Statistical analysis performed involved analysis of variance (ANOVA) with Bonferroni post hoc test. Pearson correlation coefficient analyses were performed using ImageJ Version 1.53e and JaCop plugin.

## Supplementary Material

Supplemental Material

## Data Availability

The authors confirm that the data supporting the findings of this study are available within the article [and/or] its supplementary materials.
