# Supplementary figures and images for: Enteropathogenic Escherichia coli regulates host-cell mitochondrial morphology
Source: Gut Microbes. 2022 Dec 8;14(1):2143224. doi: 10.1080/19490976.2022.2143224 (PMC9733699; doi:10.1080/19490976.2022.2143224)

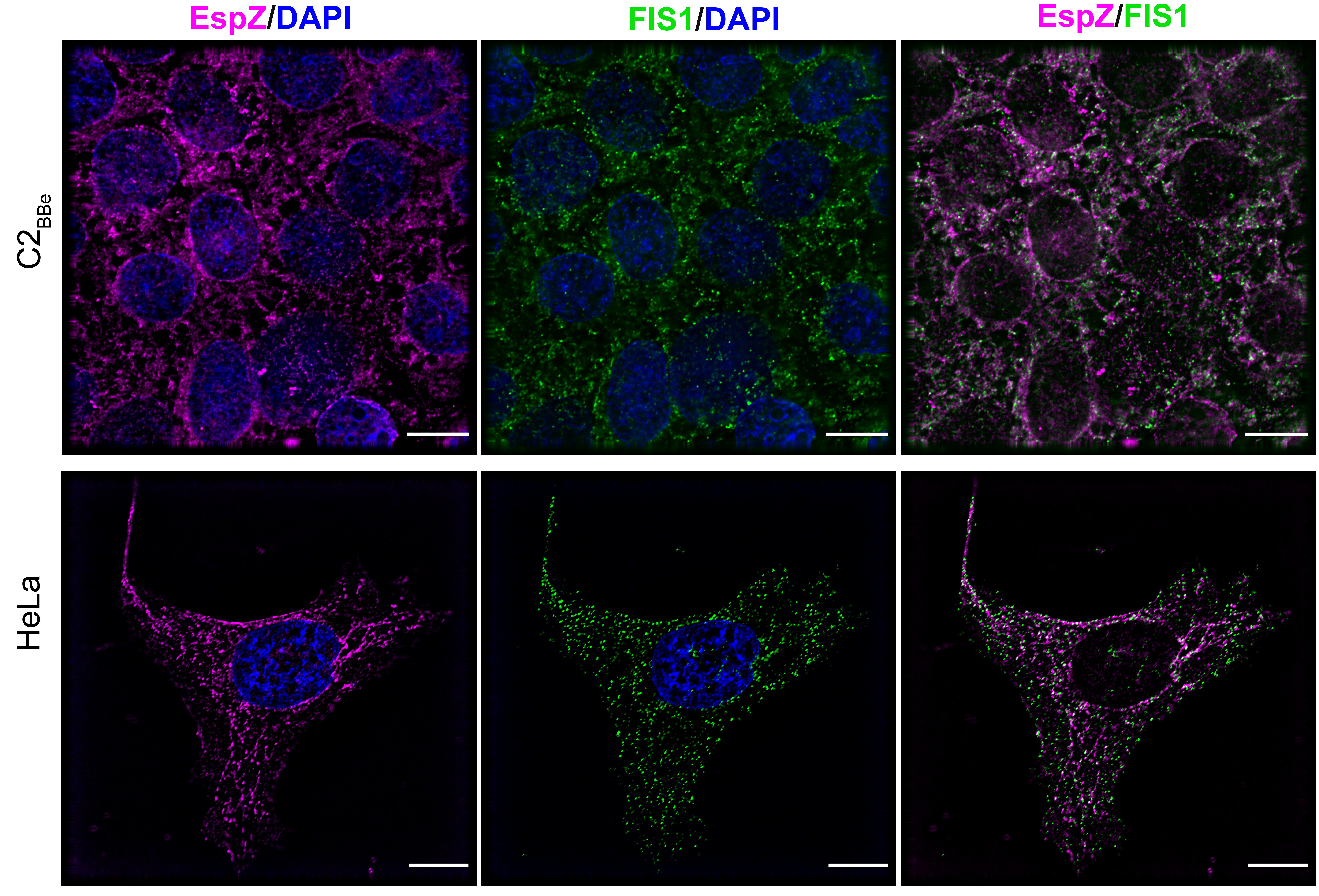

Supplement: Supplemental Material [file KGMI_A_2143224_SM8761.zip › Figure_2 (1).tif]

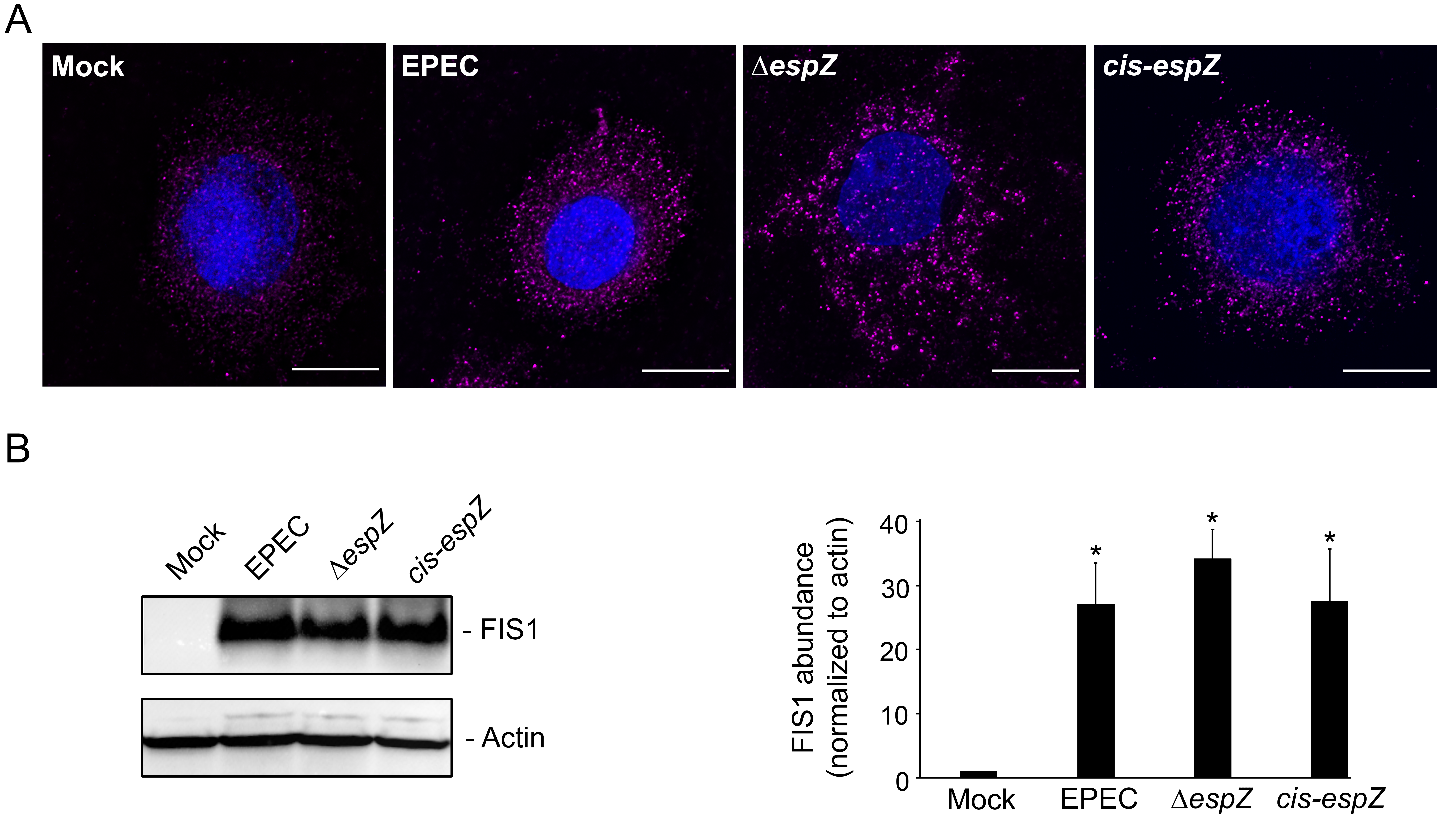

Supplement: Supplemental Material [file KGMI_A_2143224_SM8761.zip › Figure_3.tif]

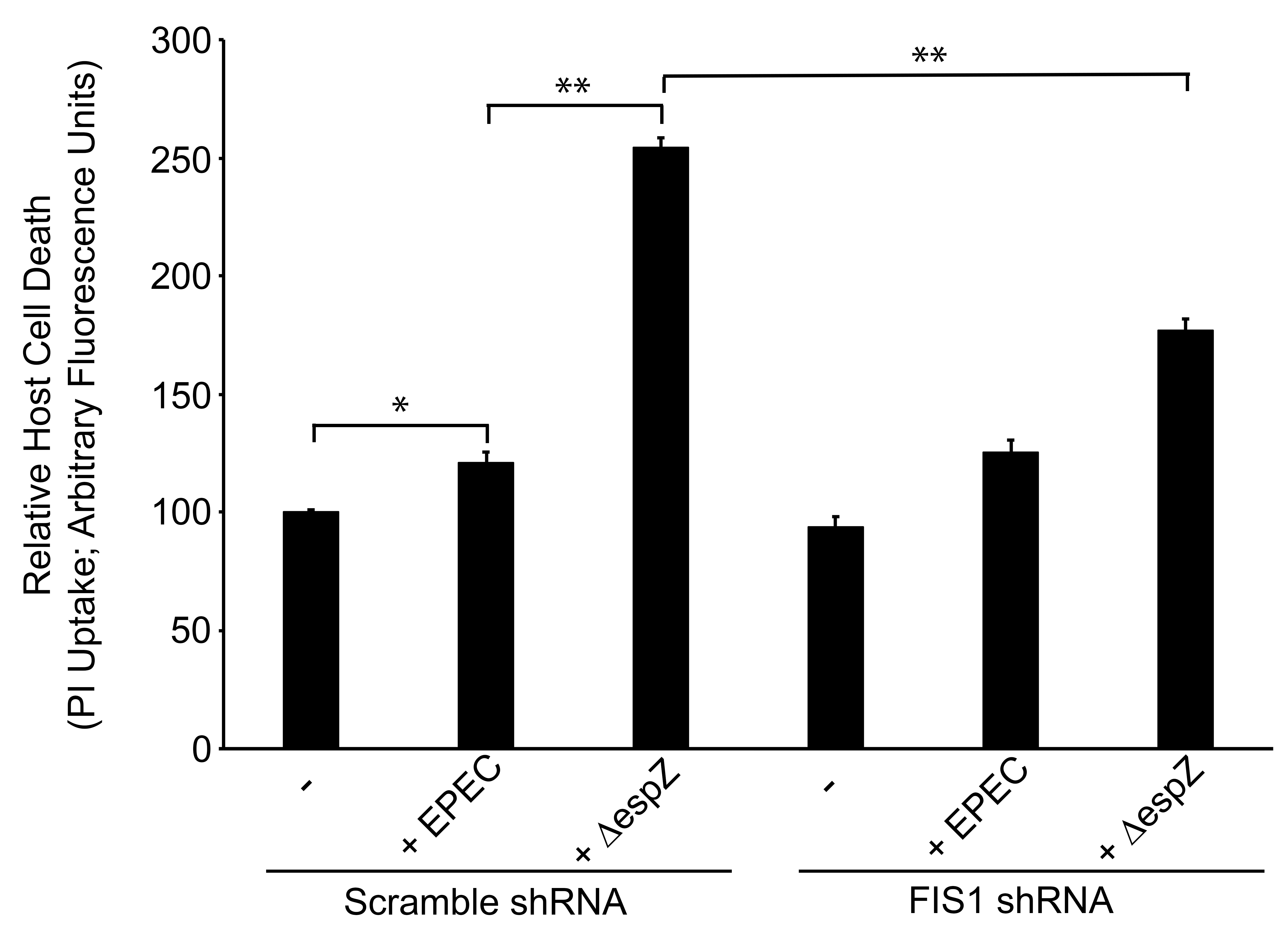

Supplement: Supplemental Material [file KGMI_A_2143224_SM8761.zip › FIgure_8.tif]

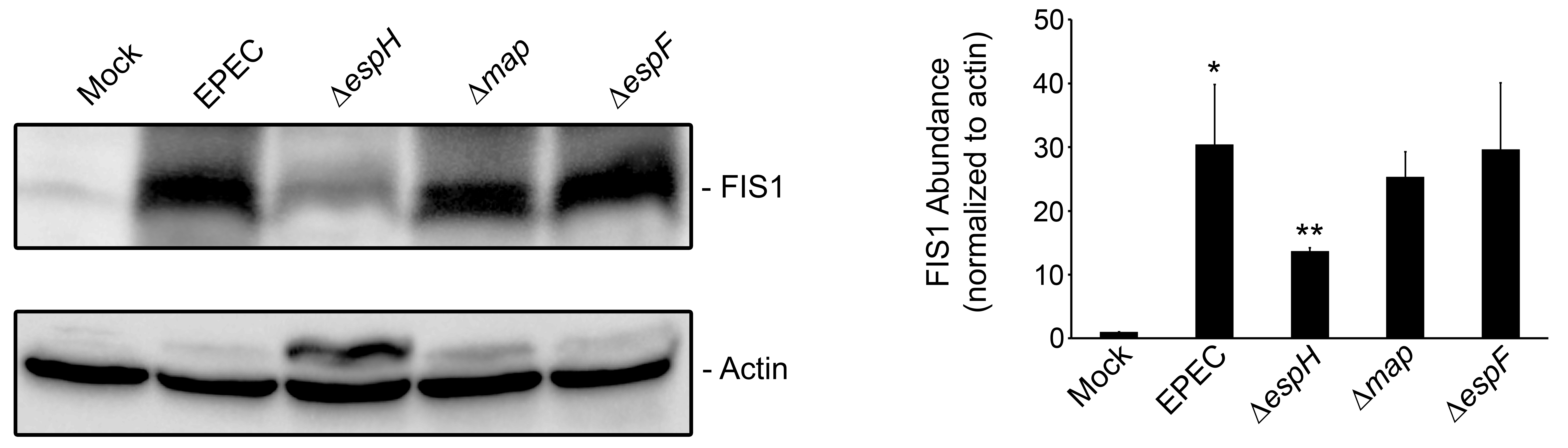

Supplement: Supplemental Material [file KGMI_A_2143224_SM8761.zip › Supplemental_Figure_3.tif]
